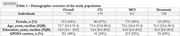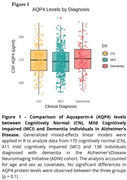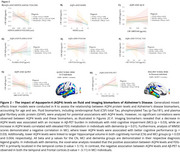# Stage‐dependent associations between CSF aquaporin‐4 levels and Alzheimer's Disease abnormalities

**DOI:** 10.1002/alz70855_104778

**Published:** 2025-12-24

**Authors:** Gabriela Mantovani Baldasso, Christian Limberger, Rodrigo Sebben Paes, Andrea Benedet, Eduardo R. Zimmer

**Affiliations:** ^1^ Universidade Federal do Rio Grande do Sul, Porto Alegre, Rio Grande do Sul, Brazil; ^2^ Institute of Neuroscienace and Physiology, University of Gothenburg, Mölndal, Västra Götaland, Sweden; ^3^ Brain Institute of Rio Grande Do Sul, PUCRS, Porto Alegre, RS, Brazil; ^4^ McGill Centre for Studies in Aging, Montreal, QC, Canada

## Abstract

**Background:**

Aquaporin‐4 (AQP4) is a water channel protein expressed by glial cells, mainly at the astroglial endfeet near blood vessels and surrounding glutamatergic synapses. Alterations in astrocytic AQP4 under pathological conditions are known to disrupt water balance regulation between the blood and the brain. However, it remains unclear whether such alterations can impact brain metabolism by disrupting glutamatergic synapses. This is particularly relevant in Alzheimer's disease (AD), where abnormal glutamate levels can lead to neuronal death and memory loss. This study investigated the association between cerebrospinal fluid (CSF) AQP4 levels, brain metabolism, and AD biomarkers.

**Method:**

Data were obtained from 719 individuals from the ADNI cohort at baseline, including 170 cognitively unimpaired (CU) individuals, 411 with mild cognitive impairment (MCI), and 138 with dementia (Table 1). Generalized linear mixed models were applied to examine the association between CSF AQP4 protein levels and AD biomarkers, adjusting for age and sex. Additionally, voxel‐wise analyses investigated the association of AQP4 CSF levels with FDG‐ and Aβ‐PET.

**Result:**

No significant differences in CSF AQP4 levels were observed between all groups (Figure 1). In CU individuals, CSF AQP4 levels were negatively correlated with hippocampal volume (b=‐0.19). Among those with MCI, CSF AQP4 levels were negatively associated with Aβ‐PET (b=‐0.11), MMSE scores (b=‐0.11) and hippocampal volume (b=‐0.17). The voxel‐wise analysis demonstrated the association between Aβ‐PET and AQP4 levels is localized in the temporal and frontal cortices. In the dementia group, CSF AQP4 levels were positively associated with FDG‐PET (b=0.24), primarily in the temporal cortex, as demonstrated by voxel‐wise analysis. No significant associations were found between CSF AQP4 levels and CSF total Tau, *p*‐Tau181, or plasma GFAP (Figure 2C‐F).

**Conclusion:**

The findings highlight AQP4 association with hippocampal volume and brain metabolism. A potential biological interpretation is that AQP4 may serve a protective function in maintaining cerebral water homeostasis, offering valuable insights into cognitive decline and potential biomarkers for AD. By facilitating these processes, AQP4 might contribute to better cognitive outcomes. However, further research is required to confirm these putative mechanisms.